# Quantum Sensing Unravels Antioxidant Efficacy Within PCL/Matrigel Skin Equivalents

**DOI:** 10.1002/smll.202403729

**Published:** 2024-09-09

**Authors:** Xixi Wu, Marcus Koch, Felipe P. Perona Martínez, Romana Schirhagl, Małgorzata K. Włodarczyk‐Biegun

**Affiliations:** ^1^ Department of Biomedical Engineering University Medical Centre Groningen and University of Groningen Ant. Deusinglaan 1 Groningen 9713 AV The Netherlands; ^2^ INM – Leibniz Institute for New Materials Campus D2 2 66123 Saarbruecken Germany; ^3^ Polymer Science Zernike Institute for Advanced Materials University of Groningen Nijenborgh 4 Groningen 9747 AG The Netherlands; ^4^ Biotechnology Centre The Silesian University of Technology Krzywoustego 8 Gliwice 44‐100 Poland

**Keywords:** antioxidant evaluation, fluorescent nanodiamond, melt electrowriting, relaxometry measurements, skin tissue engineering

## Abstract

Skin equivalents (SE) that recapitulate biological and mechanical characteristics of the native tissue are promising platforms for assessing cosmetics and studying fundamental biological processes. Methods to achieve SEs with well‐organized structure, and ideal biological and mechanical properties are limited. Here, the combination of melt electrowritten PCL scaffolds and cell‐laden Matrigel to fabricate SE is described. The PCL scaffold provides ideal structural and mechanical properties, preventing deformation of the model. The model consists of a top layer for seeding keratinocytes to mimic the epidermis, and a bottom layer of Matrigel‐based dermal compartment with fibroblasts. The compressive modulus and the biological properties after 3‐day coculture indicate a close resemblance with the native skin. Using the SE, a testing system to study the damage caused by UVA irradiation and evaluate antioxidant efficacy is established. The effectiveness of Tea polyphenols (TPs) and L‐ascorbic acid (Laa) is compared based on free radical generation. TPs are demonstrated to be more effective in downregulating free radical generation. Further, T1 relaxometry is used to detect the generation of free radicals at a single‐cell level, which allows tracking of the same cell before and after UVA treatment.

## Graphical abstract

Skin equivalents (SE) that recapitulate biological and mechanical characteristics of the native tissue are promising platforms for assessing cosmetics and studying fundamental biological processes. Here, a combination of melt electrowritten PCL scaffolds and cell‐laden Matrigel is proposed to fabricate SE. Using developed SE, a testing system to study the damage caused by UVA irradiation and evaluate antioxidant efficacy is established.

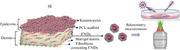

## 1 Introduction

Skin, composed of the epidermis, dermis, and subcutaneous layer, is the largest human organ and constitutes a physical barrier againsthazardous environmental stimuli.^[^ ^1^ ^]^ Aging is associated with a progressive decline in the skin's physiology, morphology, and functionality.^[^ ^2^, ^3^, ^4^, ^5^ ^]^ Age‐related damage to the skin is amplified by exposure to exogenous factors like smoking, ultraviolet radiation from the sun, and harmful chemicals from the environment.^[^ ^6^ ^]^ The primary features include the loss of skin firmness and the development of wrinkles, followed by the risk of serious diseases including skin cancer.^[^ ^7^ ^]^

Among the risk factors, ultraviolet exposure is common and difficult to avoid, resulting in oxidative damage to the skin.^[^ ^8^ ^]^ Solar UV rays are categorized into UVC (100–290 nm), which is blocked by the ozone layer, UVB (290–320 nm, constituting 5–10% of total UV), and UVA (320–400 nm, making up 90–95% of total UV), which both penetrate the ozone layer.^[^ ^9^ ^]^ UVB rays are absorbed directly by the epidermis, leading to DNA damage in keratinocytes,^[^ ^10^ ^]^ while UVA rays reach the deep dermis and cause free radical generation. These radicals induce oxidative stress, resulting in DNA damage akin to that caused by UVB, including DNA strand breaks.^[^ ^11^ ^]^ The ability of UVA to penetrate deeply and its higher proportion make it an especially harmful factor, the detrimental effects of which have long been underestimated.^[^ ^12^, ^13^ ^]^ Given this fact, it is worthwhile to prioritize research focused on understanding and reducing UVA damage to the skin. To study UVA damage, developing biomimetic in vitro skin models^[^ ^14^, ^15^, ^16^ ^]^ is an attractive alternative to testing on animals or volunteers. Numerous existing engineered models have limitations in their structure and complexity. Consequently, they do not accurately reproduce the characteristics of native human skin.^[^ ^15^ ^]^

In that context, hydrogel‐based 3D SEs are increasingly attractive as skin replacements. Hydrogels offer favorable environments for fibroblast proliferation and attachment. SEs can be constructed using only hydrogel‐based materials (e.g., decellularized extracellular matrix),^[^ ^17^, ^18^ ^]^ de‐epidermized dermis (explant),^[^ ^19^, ^20^, ^21^, ^22^ ^]^ or 3D highly‐porous scaffolds obtained from polymeric materials.^[^ ^23^ ^]^ With the help of plastic scaffolds, cells can be oriented and homogenously dispersed.^[^ ^24^ ^]^ In this way, efficient and reproducible SEs with predefined dermal thickness and cell positioning can be generated.^[^ ^15^ ^]^

Synthetic polymers with desirable biocompatibility derived from non‐natural sources offer advantages over natural ones for scaffold fabrication. They reduce the risk of pathogen contamination and zoonotic diseases, while also providing usually improved mechanical and physicochemical properties.^[^ ^25^, ^26^ ^]^ Poly(caprolactone) (PCL), poly(ethene glycol) (PEG), poly(vinyl alcohol) (PVA), poly(lactic acid) (PLA), and polystyrene were reported to fabricate skin scaffolds.^[^ ^26^, ^27^, ^28^, ^29^, ^30^ ^]^ Among those, the most commonly used polymer for tissue engineering, in general, due to its biocompatibility, superior mechanical properties, and processability is PCL.^[^ ^31^, ^32^ ^]^ To replicate the tissue microarchitecture, PCL was processed into scaffolds using such techniques as solution electrospinning, melt electrospinning, and melt electrowriting (MEW).^[^ ^33^ ^]^ MEW is a burgeoning and solvent‐free additive manufacturing technique providing high printing resolution. It allows controlled deposition of microscale fibers using specific voltage and collecting speed. MEW was utilized for different biomedical applications,^[^ ^34^ ^]^ including skin engineering. Hewitt et al.^[^ ^35^ ^]^ blended PCL with milk protein (MP) in order to enhance the biological functionality of PCL. They used MEW to print MP/PCL constructs, aiming to achieve skin regeneration by layer‐specific seeding of human keratinocytes (HaCaTs) and normal human dermal fibroblasts. However, the large pore sizes (300–500 µm) of the scaffolds resulted in difficulties in maintaining cells at precise positions, and the penetration of HaCaTs into the dermis layer was uncontrollable. As a result, the stratified structure was not ideally achieved. Afghah et al.^[^ ^36^ ^]^ introduced a hybrid scaffold combining a gelatin‐hydrogel matrix and 3D MEW‐PCL and MEW‐PCL‐Bioglass meshes. Various growth factors, including basic fibroblast growth factor (bFGF) and vascular endothelial growth factor (VEGF), were integrated into a gelatin hydrogel. The purpose was to enhance the efficiency of wound healing in combination with well‐organized PCL support. However, the functional and efficiently produced SEs are still limited and there is a vast unexplored territory in skin model research.

One of the important skin models’ applications is to investigate antioxidants that counteract reactive oxygen species (ROS) induced by UVA exposure for the development and validation of skin‐related products. For the detection of ROS, several methods exist, including quantitative polymerase chain reaction. This method detects specific enzymes generated by cells to neutralize free radicals. Its disadvantage is the lack of spatial information or single‐cell resolution.^[^ ^37^ ^]^ An alternative method makes use of organic dyes that can react with ROS and generate fluorescent signals.^[^ ^38^ ^]^ However, fluorescent dyes suffer from bleaching. In this context. fluorescent nanodiamonds (FNDs) provide a promising alternative to localize, quantify, and identify free radicals at the nanoscale.^[^ ^39^, ^40^, ^41^, ^42^, ^43^ ^]^ (NV) centers within FNDs can be used to sense free radical generation. NV centers change their optical properties based on their surrounding and thus convert magnetic signals into optical signals.^[^ ^37^, ^44^, ^45^ ^]^ The T1 relaxation measurement, provides a readout of the time needed for the relaxation, which is equivalent to T1 in conventional magnetic resonance imaging (MRI) but at a nanoscale.^[^ ^37^, ^44^, ^45^ ^]^ The T1 relaxation time is shortened by the presence of spin noise from free radicals, allowing their detection.^[^ ^46^ ^]^

**Figure 1:**
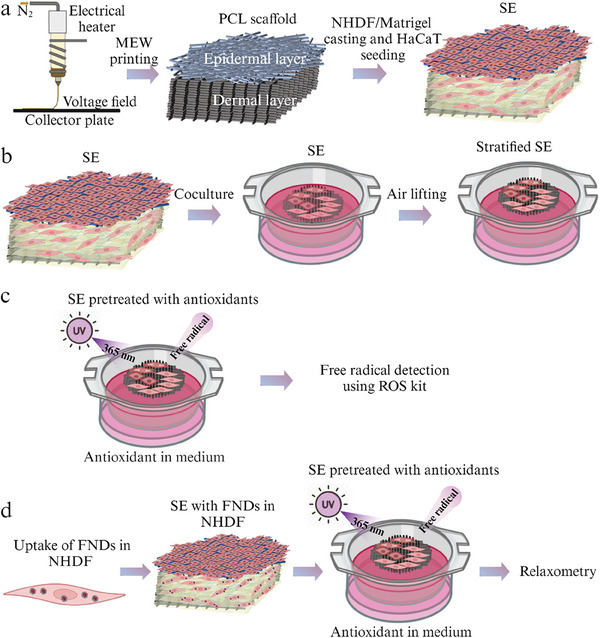
Fabrication of SE. a) A PCL scaffold was printed by MEW, Human adult dermal fibroblasts (NHDF)/Matrigel suspension was cast in the printed dermal layer, followed by keratinocyte seeding on the epidermal layer composed of random fibers. b) After 3‐day coculture in inserts, the SE was lifted to the air to achieve stratification. c) SE was irradiated with UVA and stress response was detected by ROS kit. d) FND uptake was carried out in NHDF cells and SE with FNDs was fabricated. T1 measurements were performed on the SE, and free radical production in single cells was quantified by the fitting and calculation of the T1 relaxation time. Figure 1 was generated by Biorender, the scientific illustration software (Canada), and the publishing certificate was obtained.

In our study, SEs with a comparable compressive modulus to native skin were fabricated by combining cell‐laden Matrigel with MEW‐printed PCL scaffolds (**Figure** **1**a,b). Afterward, we validated their applicability in determining antioxidant effects. Specially, we simulated the UVA irradiation of the skin and compared the efficacy of two common antioxidants, tea polyphenol, and L‐ascorbic acid, in inhibiting and scavenging ROS production. Two methods were employed and compared: a commercially available ROS kit, and T1 relaxometry (Figure 1c,d). The obtained data indicated that FND‐based relaxometry can provide accurate information on free radical concentration in single cells in the artificial dermis.

## 2 Results and Discussion

### 2.1 Physical Properties of MEW‐Printed PCL Scaffolds

**Figure 2:**
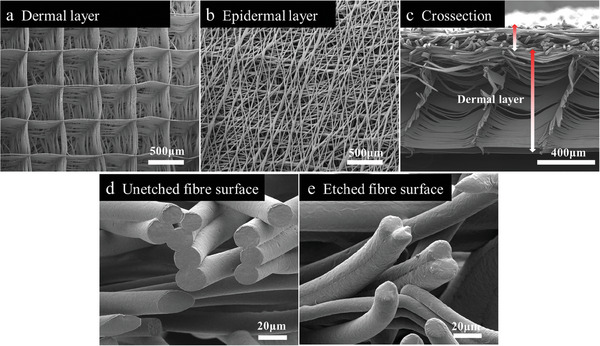
Etched PCL scaffold with two distinctive layers. a,b) Morphology of the dermal and epidermal layer, top view. Scale bars are 500 µm. c) The cross‐section of the scaffold with both layers well‐visible (the whole thickness: 0.90 ± 0.10 mm, epidermal and dermal layers labeled with the red‐and‐white arrows). Scale bar: 400 µm. d,e) Images of unetched and alkali‐etched fiber surfaces under 2000x magnification. Scale bars: 20 µm.

Multilayer scaffolds have gained considerable attention for their ability to mimic the complex and hierarchical environment of the native skin.^[^ ^47^, ^48^, ^49^ ^]^ Therefore, here, to closely mimic human skin, a two‐layer scaffold was printed with distinct porosities and architectures. Specifically, we used a well‐aligned square dermal structure and a randomly deposited epidermal layer (see **Figure** **2**). The dermal layer (Figure 2a) was composed of 128 fibrous layers, with a fiber diameter of ≈14 µm, and a pore size of 500 µm. The epidermal layer was composed of randomly oriented fibers leading to pore sizes from a few µm to 100 µm, and fiber sizes ranging from 10 to 17 µm. This dense random design was chosen to support keratinocyte seeding (shown in Figure 1a) and avoid the migration of keratinocytes into the dermal zone. After printing, the MEW meshes were subjected to alkali etching for increased hydrophilicity and, consequently, improved Matrigel penetration. There was no deformation observed in the entire construct, and both fiber diameters and pore sizes remain relatively unchanged after etching (Figure 2a,b). Additionally, a large number of fibers in the epidermis zone was still fused (see Figure 2b), yet, fibers exhibit a slight change in surface morphology (illustrated in Figure 2e). Water contact angle measurements were performed to measure the hydrophilicity of the scaffolds. The reduction of the angle from 128.33 ± 1.35° (unetched) to 30.06 ± 1.01° (etched) revealed the increased wettability of the scaffold (Figure S4, Supporting Information). This result aligns with prior studies employing alkali etching for improving the hydrophilicity of PCL scaffolds.^[^ ^50^, ^51^ ^]^ The cage‐like open structures in the dermis layer, and the improved hydrophilicity of the scaffold after etching benefited the complete penetration of Matrigel solution to obtain composite material for further studies. The thickness of the entire scaffold, measured from the cross‐section image (Figure 2c), is 0.9 ± 0.10 mm. This is very close to human skin thickness, which typically is ca. 1.1–3.1 mm, encompassing both the epidermis and dermis layers. After the addition of Matrigel, the thickness of the composite further increased to 1.0 ± 0.15 mm and fell in the range of human skin.

The porosity and density of PCL scaffolds, which generally affect the incorporation of Matrigel, gas diffusion, nutrient supply, and waste removal, were measured. The calculated porosity was ≈89%, and the density of the PCL scaffolds was 0.12 ± 0.01 g/cm^3^. According to a previous study, the preferred porosity of scaffolds for cell penetration is in the range of 60–90%.^[^ ^52^ ^]^ The porosity of scaffolds printed here, falls within this preferred range, which would be beneficial for cell penetration. However, it has been reported that NHDF cells proliferate less on large pores (>20 µm) and prefer to attach to individual fibers rather than spread across the pores.^[^ ^52^ ^]^ Additionally, cell migration distance can be increased on low‐density (<0.001 mg cm^−^^3^) and high‐porosity (>86%) scaffolds. The high density of our scaffolds might limit NHDF cell migration to other layers.

### 2.2 Fabrication and Characterization of Composite Scaffolds

**Figure 3:**
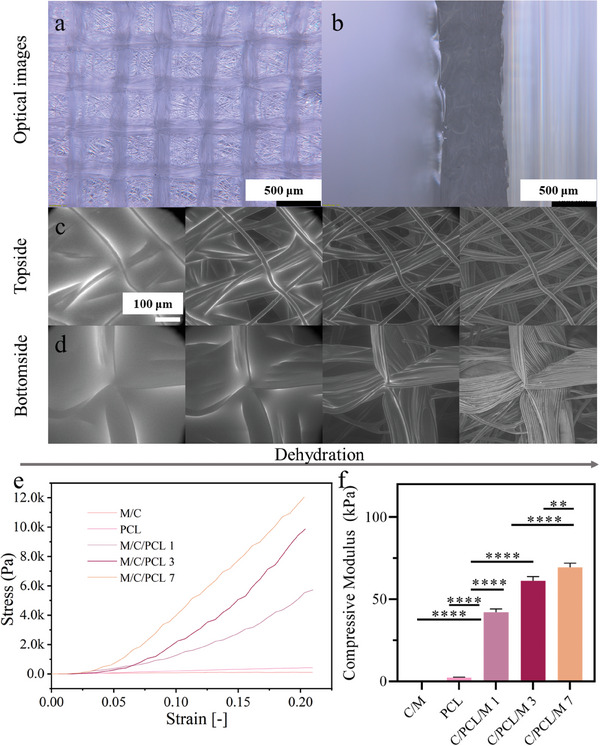
PCL‐Matrigel hybrid scaffolds a–d) Optical microscopy and ESEM. a) top view and b) cross‐section of the hybrid scaffolds. Scale bars: 500 µm. c) Topside and d) bottom side of the composite scaffolds during the dehydration process. Scale bars: 100 µm. e) Representative graphs of stress‐strain relations in compression observed for cellular Matrigel (M/C), pure PCL scaffolds (PCL), and cellular PCL/Matrigel composites on days 1, 3, and 7′s cultures (M/C/PCL 1, M/C/PCL 3 and M/C/PCL 7). f) Young's modulus calculated from the strain/stress relationship. 3–5 samples of each experimental group were evaluated, and statistical significance was done using the ANOVA test, with ^****^ indicating significant differences (*p* < 0.0001).

To obtain compressive modulus matching the native skin, we combined PCL and Matrigel matrix to create a composite scaffold. To show the distribution of Matrigel in PCL scaffolds, optical and in situ ESEM images during drying were collected (see **Figure** **3**). The optical images indicate the homogeneous integration of the PCL scaffold and Matrigel (Figure 3a,b), with the red color originating from the phenol red in Matrigel (Figure S2, Supporting Information). The ESEM images of the samples (from wet to dry state in situ drying) also confirmed the even distribution of Matrigel in PCL scaffolds from both sides (Figure 3c,d). There was no uncovered space or air pockets observed, also no deformation of PCL scaffolds after the hydrogel addition. These observations of the penetration of hydrogel into the PCL scaffold and the successful combination of materials, are supported by similar findings of Castilho et al.^[^ ^53^ ^]^

The degradation test was conducted to evaluate the stability of the PCL/Matrigel composites. The weight loss was summarized in Figure S6d (Supporting Information). Slight degradation (<5 wt%) was observed within 1 week of incubation at 37 °C, indicating the high stability of the PCL/Matrigel composites during the incubation process. The swelling ratio (Figure S6e, Supporting Information) was measured to investigate the capability of the PCL/Matrigel composites to support tissue regeneration. The high swelling ratio (>150%) of the composites (ca. 1000%) indicates a significant capability for tissue regeneration, as high swelling ratios facilitate efficient nutrient and metabolite transfer.^[^ ^54^ ^]^

To explore the mechanical properties of our skin model, the compression moduli of cell‐laden Matrigel, and cell‐laden PCL/Matrigel composite at days 1, 3, and 7 of cell culture were measured. The abbreviated names of the different cellular and acellular samples are shown in **Table** **1**. The cellular Matrigel and pure PCL constructs exhibited a compressive modulus of 0.34 ± 0.2, and 2.38 ± 0.5 kPa, respectively (Figure 3e,f). For C/PCL/M 1, a significant increase of compression moduli up to 42.1 ± 2.0 kPa was found. Visser et al.^[^ ^55^ ^]^ and Castilho et al.^[^ ^53^ ^]^ also showed that the compression moduli increased by 54 folds and 47 folds respectively, when GelMA was incorporated into PCL scaffolds. The increase in the stiffness of the composites can be attributed to the prevention of scaffold structural buckling by the surrounding hydrogel,^[^ ^53^ ^]^ and the air in pores of PCL scaffolds replaced by Matrigel. Besides, compared to cellular composites at day 1, the salient increase of compression modulus after 3‐day and 7‐day coculture was also observed (61.35 ± 2.5, 69.6 ± 2.4 kPa, Figure 3e,f), which falls within the typical compression range of human skin from 60 to 400 kPa.^[^ ^56^ ^]^ These results may be explained by the deposition of ECM produced by skin cells and by the cell‐ECM interconnections.^[^ ^57^, ^58^ ^]^ To explore ECM deposition by NHDF in Matrigel after 3 and 7 days of culture, we characterized the secretion of collagen I, the main ECM component, through immunostaining. The results, shown in Figure S3a (Supporting Information), reveal collagen I expression at both time points, with a marked increase on day 7. This difference correlates with the significant rise in the compressive moduli of the cellular PCL/Matrigel composites from 3 days to 1 week of culture. Our findings align with previous studies. Gao et al. ^[^ ^59^ ^]^ reported that after 21 days, the compressive modulus of cellular PEG/GelMA after osteogenic differentiation increased 2 folds compared to acellular PEG/GelMA scaffolds, and 1.63 folds after chondrogenic differentiation. The obtained results affirm the suitability of our composites for skin tissue engineering and regeneration.

**Table 1:** The abbreviated names of the samples.

Sample name	Sample composition	Time of cell culture [days]
C/M	Cellular Matrigel	1
PCL	PCL scaffolds	N/A
M/C/PCL 1	cellular PCL/Matrigel composites	1
M/C/PCL 3	cellular PCL/Matrigel composites	3
M/C/PCL 7	cellular PCL/Matrigel composites	7

To obtain the cell morphology information of keratinocytes and fibroblasts, cell F‐actin and nuclei were stained. As visible in Figure S5c,d (Supporting Information), spindle‐shaped fibroblasts were present in the dermal compartment, and well‐spread keratinocytes were observed in the epidermal layer.

### 2.3 Hematoxylin and Eosin (H&E) and Immunohistological Staining on SEs

**Figure 4:**
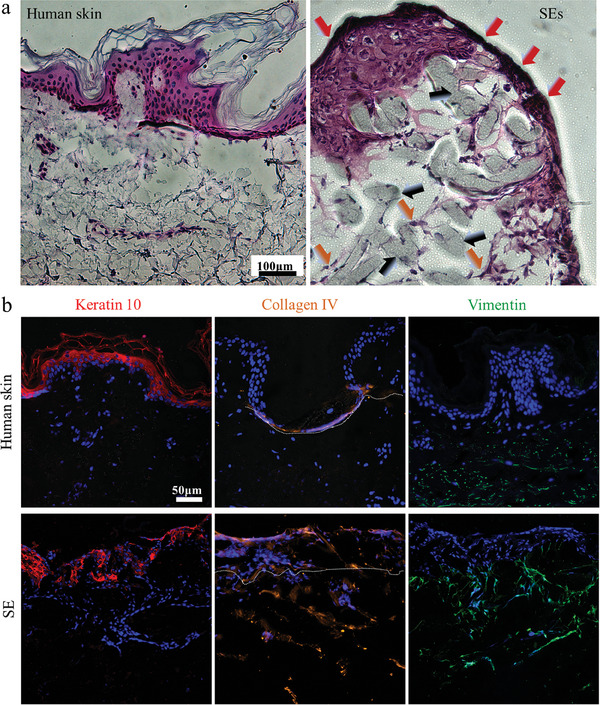
Native skin (control) and SE staining. a) H&E staining images of section slices after 3‐day cell culture. The cell nuclei are shown in purplish‐blue, while cytoplasmic components are stained in pink, the images were adjusted in brightness (−20%) and contrast (+40%) to improve clarity. Scale bars: 100 µm. b) Biomarker analysis: Cell nuclei are stained with DAPI (blue), epidermal layers are labeled by keratin 10 (red), dermal layers are labeled by vimentin (green), and cell junction parts are marked by collagen IV (orange). Scale bars: 50 µm. Five skin replicates were cryosectioned, and 10 sections of each replicate were stained and imaged.

To confirm the formation of a SE, the skin slices were analyzed by H&E and immunohistological staining. The H&E staining images confirmed that the epidermis and the dermis were well‐formed after 3‐day coculture (**Figure** **4**a), and the thickness of the epidermis and dermis in SE samples was similar to native skin. The interface between the epidermis and dermis was also clear. The structure including the stratified layer (marked with red arrows in Figure 4a‐“SE”) of the SE was comparable to the native human skin. The sections of the PCL scaffold are highlighted with black arrows (Figure 4b). In the artificial epidermis, several layers of living cells could be identified, and the cytoplasmic components were well‐organized compared to human skin. The fibroblasts formed spindle shapes in the artificial dermis (marked with orange arrows in Figure 4a‐“SE”), similar to the real dermis. The histological analysis confirms the successful skin formation utilizing the composite scaffolds.

To obtain further information on the obtained stratified SE, i.e., identify the location of cells and the expression of biomarkers in skin cells, the section slices of the samples were stained for nuclei, keratin 10 (a marker for HaCaTs), collagen IV (an indicator of epidermis‐dermis conjunction part), and vimentin (a marker for NHDF cells, shown in Figure 4b). The secretion of keratin 10 expressed in keratinocyte layers was shown in the suprabasal compartment, visible in our models and real skin. The conjunction region of epidermis and dermis, as an important feature of skin tissue, is characterized by the expression of collagen IV.^[^ ^16^, ^60^ ^]^ The presence of collagen IV (highlighted with white dashes in the “Collagen IV” column in Figure 4b) in human skin and SE indicated the well‐connected epidermis and dermis. The background noise in the collagen IV staining originates from the Matrigel, which also contains collagen IV. The uniform expansion and alignment of spindle‐shaped fibroblasts were revealed in the immunostaining images of vimentin (Figure 4b “Vimentin” column). Some fibroblast cells were situated near the region beneath the epidermis, similarly to the human skin. In short, biomarker labeling in the epidermal layers, conjugation region, and dermal layers indicated the successful formation of a SE, offering a promising prototype for future skin testing.

Cell penetration depth was studied to evaluate the potential infiltration inside the melt electrowritten structure during 3‐day and 7‐day coculture. The representative images of the keratinocyte layer labeled with cytokeratin 10 antibody (red, Figure S7a, Supporting Information) reveal no keratinocyte penetration into the dermal layer, as the structure here consists of densely packed random fibers. The penetration depth of fibroblasts into the dermis layer was ≈700 µm, which closely mimicked the native skin (ca. 700 µm) (see Figure S7b,c, Supporting Information). The dermis layer, with relatively large fiber distances (500 µm) and filled with cell‐laden Matrigel, ensured the homogeneous distribution and penetration of cells throughout the entire engineered dermis layer.

Further, when grown in Matrigel, there was no significant difference in cell metabolism between 3‐day and 7‐day cultures as analyzed with the Alamarblue cell metabolism kit (Figure S3b, Supporting Information). The similar cell numbers and metabolic activity indicate little cell proliferation by extending the culture time from 3 days to 1 week. Additionally, no obvious cell migration was observed between the epidermis and dermis within 1‐week coculture in PCL/Matrigel composites. Thus, a 3‐day coculture was utilized to do the following measurements.

### 2.4 Evaluation of the Antioxidant Effect: the Ability to Decrease ROS Concentration under UVA Irradiation

To exemplify the applicability of our model, UVA irradiation was utilized to induce oxidative stress, and the protective effect of antioxidants was studied. The produced ROS was measured by ROS kit and free radical production by T1 relaxometry.

#### 2.4.1 Detection of ROS Using the Commercial Kit

**Figure 5:**
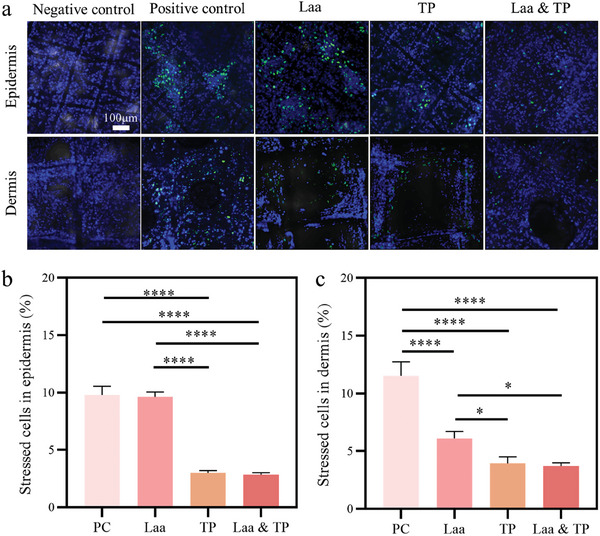
a) Fluorescent staining of SE showing the production of ROS in epidermal and dermal cells under UVA radiation. The SEs were treated with Laa, TP, Laa&TP, and the SE with cell medium without UVA irradiation was used as a negative control, the corresponding samples in cell medium under UVA irradiation were used as the positive control. The cell nuclei stained in blue, and stressed cells stained in green. Scale bars: 100 µm. b,c) Ratios of stressed cells in epidermal and dermal layers. The experiment was repeated 3 times, and error bars represent the standard deviations. Statistical comparisons were made using the ANOVA test, with ^*^, and ^****^ indicating significant differences (*p* < 0.05, *p* < 0.0001, respectively).

To analyse the antioxidant activity against UVA exposure in SE, the production of ROS was measured. In the absence of UVA exposure (negative control), the SE exhibited few stressed cells (labeled with green fluorescence, Figure S5, Supporting Information). However, after UVA radiation, damage caused by oxidative stress on cells was detected (**Figure** **5**a‐PC). The scavenging activities were evident for both antioxidants when compared to the positive control groups (no antioxidant, UVA exposure; PC in Figure 5a). In detail, TPs exhibited a superior capability to remove ROS in both the epidermal and dermal layers. This is indicated by the lower ratio of oxidative stress in cells compared to the Laa group (Figure 5a–c). Additionally, in the epidermis, no significant difference was observed between the PC and Laa‐treated groups. This indicates the low activity of Laa in protecting the epidermis (Figure 5a,b). However, after Laa treatment, a lower ratio of cells producing ROS was observed in the dermal compartment (Figure 5a,c), revealing the protective effect of Laa in the deep dermis. L‐ascorbic acid may have better penetration into the dermis compared to the epidermis, thus the efficacy of Laa mainly appeared in the dermis.^[^ ^61^, ^62^ ^]^ Further, there was no statistically significant difference between TPs and “Laa & TPs” groups, indicating the scavenging effect is primarily from TPs (Figure 5).

The results can be explained by the different mechanisms of lowering the ROS concentration in cells by both antioxidants tested. UVA photons can be directly absorbed by DNA, which causes DNA lesions and the repair process recruits relevant enzymes, the ROS is generated as by‐products. Further, the photons can also be absorbed by the chromophores that are present in skin cells. This reaction with chromophores leads to the formation of ROS, resulting in oxidative stress.^[^ ^11^ ^]^ The increased stress leads to defects in mitochondria, which further perpetuate the production of ROS.^[^ ^63^ ^]^ Further, UVA radiation can enhance metal‐catalyzed reactions and lipid peroxidation, which leads to ROS formation as well. The NADPH Oxidase can be activated in the meantime, which is an enzyme responsible for generating superoxide radicals.^[^ ^64^, ^65^, ^66^ ^]^ TPs, including catechins, flavonoids, and other compounds found in tea (especially green tea), possess strong antioxidant properties.^[^ ^67^, ^68^ ^]^ Besides, Tea polyphenols induce the expression of endogenous antioxidant enzymes,^[^ ^69^, ^70^ ^]^ help prevent lipid peroxidation by stabilizing cell membranes and interfering with the chain reaction of lipid oxidation.^[^ ^71^, ^72^, ^73^, ^74^ ^]^ On the other hand, Laa which is known as Vitamin C, has relatively limited pathways to reduce ROS. Laa can donate electrons to neutralize unstable free radicals such as hydroxyl radicals (•OH) and superoxide radicals (O_2_•−).^[^ ^75^ ^]^ Similar to TPs, Laa can chelate metal ions such as iron and copper, reducing their ability to generate highly reactive hydroxyl radicals.^[^ ^76^, ^77^, ^78^ ^]^ Combining the triggers for ROS production from UVA exposure and the ROS attenuation mechanism treated by TPs and Laa, TPs ought to be more potent than Laa. TPs are effective in reducing ROS by preventing production and directly scavenging ROS. Rice‐Evans et al. reviewed the studies on the effectiveness of TPs and Laa.^[^ ^79^ ^]^ She concluded that TPs were more powerful in scavenging ROS due to contributions from catechin/gallate constituents.^[^ ^84^ ^]^ Our results align with existing research and are the first to compare the efficacy of TPs and Laa in protecting skin cells from oxidative lesions caused by common UVA radiation.

#### 2.4.2 T1 Measurements on Detecting Free Radicals in Individual NHDF Cells Situated in the Dermis

##### Characterization of Quaternized β‐chitin (QβC) Coated FNDs (C/FNDs), Diamond Uptake, and Biocompatibility

**Figure 6:**
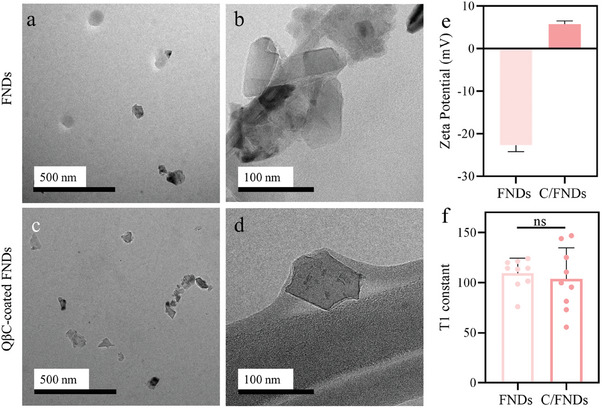
FNDs Characterizations. a,c) Cryo‐TEM images show the dispersion of pure FNDs and QβC‐coated FNDs in PBS. b,d) TEM images exhibit surface morphology of the FNDs and C/FNDs. The scale bars are shown in the figures. e) Zeta potentials before and after FND modification. f) T1 of FNDs with and without QβC layers. The statistical significance was analyzed using a T‐test, ns means no significant difference.

To determine C/FNDs morphology, particle size and zeta potential were measured. The results are shown in **Figure** **6**. After QβC coating, slight agglomerates can be observed in cryo‐TEM images, bare FNDs are shown as controls (Figure 6a,c). Zeta sizer data also confirmed slight aggregation during measurement after modifying FNDs with QβC, the measured sizes of FNDs increased from 100.25 ± 2.5 to 137.76 ± 3.7 nm (Figure S8, Supporting Information). Additionally, there is a clear coating film on single FNDs visible under higher magnification in TEM images (Figure 6d). The charges of modified FNDs were increased from −22.5 ± 1.3 to 5.8 ± 0.5 mV (Figure 6e), validating the successful coating of FND surfaces. The relatively lower zeta potentials (5.8 ± 0.5 mV) explained the slight instability of C/FNDs in PBS compared to the initial high negative charges (−22.5 ± 1.3 mV), indicating a more stable dispersion system.^[^ ^80^, ^81^, ^82^ ^]^ The pre‐evaluation of diamond relaxation time showed no significant difference between the bare and coated FNDs (Figure 6f), suggesting no compromise in detecting sensitivity after coating.

**Figure 7:**
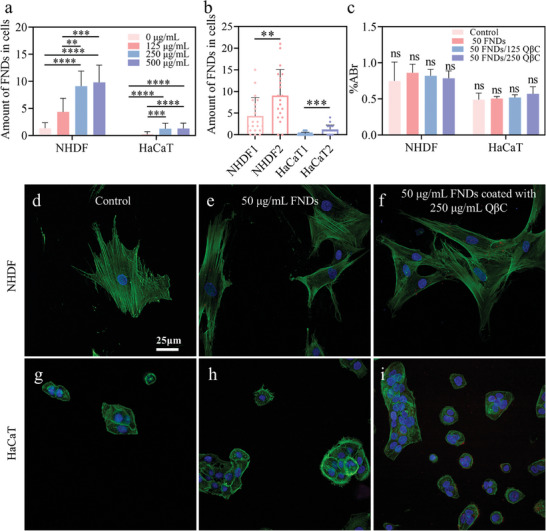
Cell uptake of bare and coated FNDs. a) The optimization of diamond uptake in both skin cells after adding 50 µg mL^−1^ FNDs with different concentrations of coating material. b) The numbers of ingested FNDs in NHDF and HaCaT cells with bare FNDs (denoted with 1) and C/FNDs (denoted with 2). Significantly increased uptake was observed with 250 µg mL^−1^ QβC and 50 µg mL^−1^ FNDs. c) Biocompatibility of FNDs tested by determining the cell metabolic activities at day 1 using an AlamarBlue kit. d–i) Fluorescent images to show FND uptake after 24 h. The cytoskeleton was stained in green, nuclei in blue, and FNDs or C/FNDs are shown as red dots. Cells without treatment were used as controls. Scale bars: 25 µm. To calculate FND uptake in cells, 20 cells of each experimental group were analyzed. For cell metabolism, three samples of each group were measured. The mean values with SD were indicated in each figure, statistical comparisons were made using the ANOVA test, “ns” means no significant difference, ^**^ indicates *p* < 0.01, ^***^ indicates *p* < 0.001, ^****^ indicates *p* < 0.0001.

During our trials on the diamond uptake, pure FNDs were barely taken up by the HaCaT and NHDF cells. We observed several FNDs found in NHDF, and few in HaCaTs (Figure S11a, Supporting Information) even at relatively high concentrations of FNDs (50 µg mL^−1^) and long incubation times (24 h). This can be explained by the charge repulsion between nanodiamonds and cell membranes. To increase FND uptake, we added positive charges to the bare FNDs by coating them with QβC. After the optimization of FND's and coated material's concentrations (Figure S11a, Supporting Information; Figure **7a**), the efficient internalization of C/FNDs can be observed in both skin cells (**Figure** 7). Specifically, compared to bare FNDs at 50 µg mL^−1^, there was a gradual increase in diamond uptake as the concentration of QβC increased (from 125 to 250 µg mL^−1^ in Figure 7a). The increase was almost 7‐fold for both cell lines with QβC at the concentration of 250 µg mL^−1^ (from 1.35 to 9.8 for NHDF and 0.2 to 1.3 for HaCaTs, shown in Figure 7b). However, there was no difference when the concentration of QβC increased from 250 to 500 µg mL^−1^. Biocompatibility of FNDs was confirmed after 1‐day cell culture based on the comparable Alamar blue reduction level shown in Figure 7c.

Therefore, the concentrations of FNDs and QβC were selected as 50 and 250 µg mL^−1^, respectively. After 1‐day incubation in the medium with FNDs and QβC, there was avisible increase in the numbers of nanodiamonds appearing in the NHDF and HaCaT cells (red dots in Figure 7f,i). Only slight aggregates were observed in the cells with the C/FNDs; these aggregates are below 1 µm in diameter (Figure 7f,i). The positive surface charges due to the coating contributed to the enhanced FND uptake in the cells. An explanation for this behavior could be the electrostatic interactions between positively charged particles and negatively‐charged cell membranes.^[^ ^83^, ^84^, ^85^ ^]^ To increase FND uptake, other types of coating were reported, including recombinant polypeptide,^[^ ^86^ ^]^ polymers,^[^ ^87^ ^]^ silica,^[^ ^88^ ^]^ nucleic acids,^[^ ^89^ ^]^ lipid,^[^ ^90^ ^]^ and dextran coatings.^[^ ^91^ ^]^ Another strategy proposed was to permeabilise the cell membrane via treatment using trypsin‐EDTA.^[^ ^92^ ^]^ However, the mentioned methods either involve complex synthesis or can be harsh to the cells, potentially disrupting their natural behavior. The relatively simple lipid‐coating and trypsin‐EDTA treatment did not result in improved uptake for our cells (Figure S11b, Supporting Information). Therefore, as proposed here QBC coating is an interesting, simple‐to‐use alternative.

##### Free Radical Detection Using T1 Relaxometry

**Figure 8:**
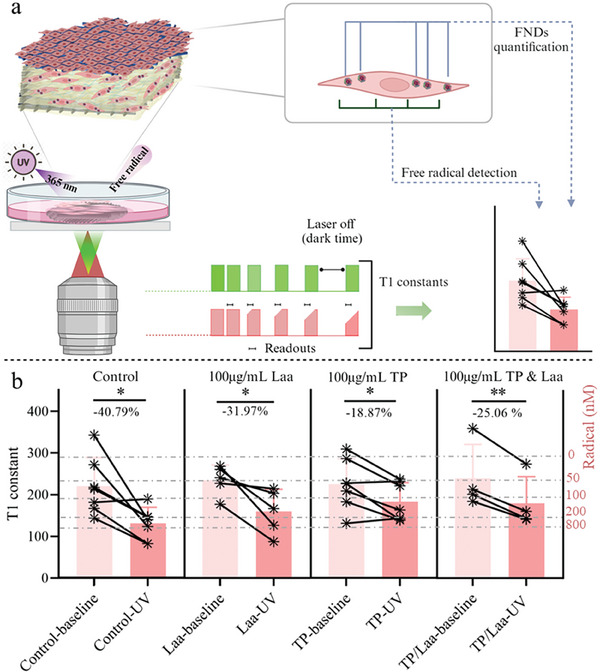
T1 measurements were conducted to measure free radical generation in the artificial dermal layer of the SE. a) A schematic summary of the experimental conditions investigated using T1 measurements. First, the FSTE was generated with NHDF containing C/FNDs, after 3‐day incubation, the SEs were incubated with 100 µg mL^−1^ TPs, Laa, TPs, and Laa respectively overnight. Free radical formation in single NHDF cells was investigated. The initial T1 values were recorded and denoted with baseline. The same nanodiamonds were tested after UVA irradiation and denoted with UV. b) Summary of T1 results for these experimental groups. The grey dashed lines represent the approximated concentration calibrated with ^*^OH radicals in the solution from the previous work.^[^ ^93^ ^]^ 4–8 samples were tested, the average values were collected and analyzed. Statistical comparisons were made using a paired t‐test. ^*^ means *p* < 0.05 and ^**^ *p* < 0.01. Figure 8 was generated by Biorender.

The abovementioned results mainly revealed the number of stressed cells. To obtain free radical concentrations at subcellular resolution, T1 relaxometry was conducted. **Figure** **8**b shows the evolution of T1 and the radical generation before and after UVA exposure, when pretreating SEs upon treatment with various antioxidants. The T1 values of samples with TP treatment were slightly lower (≈18%) compared to other experimental groups (control 40.79 ± 9.56% and Laa treatment 31.97 ± 7.23%), suggesting the relatively lower free radical generation in TPs samples. When adding both TPs and Laa, no differences in T1 were observed when compared to TPs treatment, but the T1 values of TP & Laa groups were significantly lower than in the group with only Laa. This reveals that TPs have a stronger effectiveness in reducing the free radical load. The results of the conventional assay and diamond‐based quantum sensing are in good agreement here. Additionally, the free radical production in single cells can be observed and compared (Figure 8b), which shows biological variability between single cells.^[^ ^37^ ^]^ While conventional assays are limited by parallel sample variability, these measurements are repeatable on the same individual particle/cell, allowing the detection of antioxidant efficacy and differentiation caused by interventions like UVA irradiation.

It is important to note that once ingested by cells, NHDF cells tend to excrete FNDs (over 3–5 days) due to cell mitosis and exocytosis. This complicates measurements at later time points. To mitigate this issue, we conducted T1 measurements on SEs after a 3‐day culture. The conjugation part of the SEs was created by incorporating keratinocytes and fibroblasts. While the formation of stable SEs was confirmed, the skin models may lack sufficient cell‐cell and cell‐matrix interconnections. T1 tests on SEs after long‐term coculture (7 days) were also explored. By elongating the FND incubation time with NHDF, remaining FNDs were found in NHDF cells after a 7‐day culture. A significant decrease in the T1 constant was observed in the graph (Figure S13, Supporting Information), revealing the capability of the FNDs to give the signals of free radicals after 1‐week culture. However, increasing the number of FNDs (≈18 per cell) can lead to aggregation, making it challenging to obtain clear FND signals. In our future research, we will focus on reducing FND aggregation and extending their retention time in cells to enable broader applications of FNDs in the biomedical field.

## 3 Conclusion

An SE was fabricated by incorporating fibroblast‐laden Matrigel into a multilayer hybrid PCL scaffold printed using MEW, followed by the seeding of keratinocytes on top of the construct. A favorable environment for the development of a relevat skin model was provided by this method. The resulting model mimicked the mechanical properties of the native skin. The histological analysis and immunostaining proved comparable biological properties of the model to human skin. Further, the SE was applied to study the impact of UVA skin irradiation, and the efficacy of TP and Laa antioxidants against UVA damage. By applying both, the ROS kit and T1 relaxometry, the oxidative stress in different experimental groups was measured. TPs proved to have better effectiveness in downregulating ROS production. The proposed skin model provides a useful system for ROS testing and antioxidant evaluation, indicating its applicability for drug testing or exploration of fundamental biological processes of skin tissue. Additionally, T1 measurements were proposed as a novel free radical detection technique in the skin model, enabling the quantification of free radicals at the single‐cell level.

## 4 Experimental Section

### Fabrication of MEW‐Printed PCL Scaffolds

The PCL scaffolds were printed following the optimized procedure. Specifically, 2 g polycaprolactone (PCL, Purasorb PC 12, Corbion) pellets were added into a steel cartridge customized for MEW. After fitting the 0.25 mm bronze nozzle (E3D online, UK), the cartridge was loaded into the heating cylinder of the MEW printer (Spraybase A‐1204‐0001‐01D, Ireland). The scaffold was designed with two zones, 128 layers of a well‐organized square mesh at the bottom and 40 layers of densely deposited random fibers on top. The printing temperature was maintained at 100 °C, with a 5 mm gap between the nozzle and collector. The printing velocity was set to 250 mm min^−1^, the pressure to 0.1 bar, and the voltage to 6 kV. The resulting size of the fibers was 14.8 ± 0.5 µm. The ultimate dimensions of the printed scaffold were 20 mm x 20 mm x 0.9 mm.

### Fabrication and characterization of PCL‐Matrigel scaffolds and skin replicates: Preparation of PCL‐Matrigel Scaffolds and Skin Replicates

To improve the hydrophilicity of the PCL scaffolds to provide better integration with the hydrogel, the study etched them by immersion in 5 m NaOH for 2 h. After washing with 500 mL deionized water for two days (the water was changed every 30 min at daytime), the etched PCL scaffolds were dried and sent to SEM analysis (FEI Quanta 400 FEG, the USA) for morphology and thickness investigation. For SEM imaging, a cross‐section of the sample was prepared by placing the scaffold in a metallic vessel filled with liquid ethanol, solidifying the ethanol after transfer of the vessel to a Dewar flask filled with liquid nitrogen, and cutting the scaffold vertically using a blade. After thawing the ethanol and drying the sample on a tissue, the cross‐section was mounted on the side surface of a metallic cube using double‐sided carbon tape. Gold was sputter deposited (JEOL JFC‐1300, 30 mA, 30 s) on the sample before SEM visualization in high vacuum mode using secondary electrons (Everhart‐Thornley detector) at 3 kV accelerating voltage.

The wettability of both untreated and etched scaffolds was assessed using a DataPhysics OCA30 (USA) system. Droplets of pure water (Milli‐Q), each comprising 5 µL, were deposited on the samples through an automated syringe, and subsequent images were captured. Three measurements were conducted for each sample, and the average values were reported.

The measurement of the density and porosity of PCL scaffolds, along with the weight loss and swelling ratio study on PCL/Matrigel composites, were provided in SI information.

Human epidermal keratinocytes (HaCaTs) were purchased from CLS Cell Lines Service (CLS, USA). The cells were cultured in HEPES‐buffered Dulbecco's modified Eagle medium (DMEM, high glucose, GlutaMAX Supplement, Gibco, USA), supplemented with 10% fetal bovine serum (FBS, Gibco, USA), 1 v/v % Penicillin‐Streptomycin, and 0.2 v/v% Amphotericin B. Human adult dermal fibroblasts (NHDF‐Ad; Lonza, The Netherlands) were also cultured with the same medium. All cell flasks and well plates were maintained during the culture in a 37 °C, 5% CO_2_, humidified incubator.

Matrigel (Corning, CLS356234, the USA) was diluted to 5.5% (w/v) (the concentration was kept in the following studies) with acellular medium or cell suspension. The mixture was injected into an etched sterile PCL scaffold whose size was adjusted to fit the 48‐well plates (1.1 cm^2^). The entire process was conducted in an icebox following the handbook provided by Corning for Matrigel. The cell spreading and ECM deposition in Matrigel were evaluated. After 3‐day and 7‐day cultures, the samples were fixed with 4% Paraformaldehyde (PFA) for 10 min, followed by washing with PBS three times. Then the samples were permeabilized with 0.05% triton (Sigma), after washing, the cell samples were blocked with 5 wt% Bovine Serum Albumin (BSA, Sigma) for 30 min. After removing BSA, the samples were incubated with Anti‐Collagen I antibody (1:200 in PBS, Abcam), and Anti‐Vimentin antibody (1:200, Sigma) respectively at 4 °C overnight. After washing, the cells were treated with phalloidin‐FITC (Sigma) for 45 min to label the f‐actin of the cytoskeleton. DAPI was used to stain cell nuclei. The samples were imaged by laser confocal microscopy (Leica Stellaris 5, Germany), and images were analyzed using ImageJ, the results were provided in Figure S3a (Supporting Information). The NHDF cell metabolism in 5.5% (w/v) Matrigel was evaluated. Specifically, NHDF cells were suspended in Matrigel, the final concentration was 2 × 10^5^ cells mL^−1^. 300 µL cell suspension was added into a 4‐chamber petri dish to obtain the 3D‐culture layer at ca.1‐mm thickness. The cell metabolism at days 1, 3, and 7 was measured by an Alarmablue kit following the previous study,^[^ ^94^ ^]^ the result was presented in Figure S3b (Supporting Information).

First, the dermis layer was formed, by injecting Matrigel with 100 µL 1 × 10^7^ cells mL^−1^ concentration of NHDF into the MEW printed scaffold. The thermal crosslinking was performed for 15 min in the 37 °C incubator. Next, NHDF and HaCaT were mixed in 5.5% (w/v) Matrigel and cast on the top of the dermal layer to facilitate the conjugation of epidermis and dermis, the concentration of the cells was 2.5 × 10^6^ cells cm^−2^ with a cell ratio of 1:4000 (HaCaT: NHDF). The cross‐linking of the junction part was performed for 15 min following the same procedure as for the dermal layer. Finally, HaCaTs (1 × 10^8^ cells mL^−1^) were seeded on the random fibers to form the epidermal layer. The whole casting procedure was performed from the random fiber side. Subsequently, cell medium was added around the composites, and samples were maintained in a 37 °C, 5% CO2, humidified incubator. The composites, with or without NHDF cells, were then transferred from 6‐well plates into 24‐well inserts and left for 3 days, and 7 days to compare the skin regeneration stage. In the 3‐day coculture system, 3‐day cultivation and 1‐day air‐lifting of epidermal layers were performed to achieve the fast generation of the SEs (further abbreviated as M/C/PCL 3), for 7‐day coculture, 7‐day cultivation, and 3‐day air‐lifting were performed (abbreviated as M/C/PCL 7). The morphology of cells in the composites on day 3 was observed by staining F‐actin and cell nuclei following the protocol mentioned before. The stained samples were imaged by confocal microscopy mentioned before.

### Fabrication and characterization of PCL‐Matrigel scaffolds and skin replicates: Imaging and Compression Measurements on PCL‐Matrigel Hybrid Scaffolds and Skin Replicates

Optical images were collected to evaluate the integration of PCL scaffolds and Matrigel. To visualize the dispersion of Matrigel inside the PCL scaffolds during dehydration in situ, ESEM images of the PCL‐Matrigel hybrid scaffolds were captured from both the top and bottom sides at 3 °C and 10 kV accelerating voltage. The water vapor pressure inside the ESEM chamber was changed from 750 to 350 Pa (corresponding to a change from 100% to 47% relative humidity at 3 °C).

Compression tests of the different cellular and acellular samples (see Table 1) were measured by a homemade low‐load compression tester (LLCT, the Netherlands). The applied configuration of the LLCT instrument was previously described.^[^ ^95^ ^]^ In detail, the LabVIEW 7.1 program was utilized to collect data from the LLCT load cell. The geometry moved downward at a speed of 5 µm s^−1^ until it experienced a counterforce of 10^−4^ N. Then, the deformation of the samples was set at 20% of their original thickness (strain ε  =  0.2) at a velocity of 2%/s (strain rate ε̇ = 0.2 s^−1^). The indentation probe was 2.5 mm in diameter. The deformation was kept at the same state with the applied stress monitored for 100 s. After the compression tests, the stress and the strain applied were plotted in the LabVIEW 7.1 program. A linear relationship between stress and strain was identified in the plot within the strain range of 0.05–0.1. The slope of this line, which signifies stiffness (Young's modulus), was determined, and the average values from three samples were calculated.

### Fabrication and characterization of PCL‐Matrigel scaffolds and skin replicates: Cryosection and H&E Staining, Immunohistology, and Image Analysis of Skin Models

For comparison with the artificial skin model, human skin samples were used from the abdomen. These samples were obtained from patients who underwent plastic surgery at the UMCG. Ethical considerations were cautiously addressed, with approval from the Institutional Review Board sought and subsequently exempted (Reference No. M24.332256), as the study employed anonymized waste material. More specifically, the waste material consisted of skin tissue which was removed during surgery within the standard clinical care. Necessary guidelines were followed to use these samples in the main manuscript. All participating patients provided informed consent, with their materials processed anonymously. The native skin and the artificial skin replicates (M/C/PCL 3) were fixed with 4% Paraformaldehyde (PFA) for 20 min, followed by washing with PBS 3 times. The samples were embedded in an Optimal cutting temperature Compound (O.C.T. Compound, Thermo Fisher Scientific, the USA) for 30 min and then frozen by liquid nitrogen. To observe the histological properties, the samples were sliced into 10 µm sections using a cryostat (Epredia CryoStar NX70, Japan). 5 skin replicates were prepared for cryosection and 10 sections of each replicate were stained and imaged.

The slices were immersed in PBS for 10 min to remove the O.C.T. Compound. Afterward, the sample slices were stained with hematoxylin for 2 min and washed with tap water for 5 min. In order to reduce the background from hematoxylin (Abcam, the USA), 1% acid alcohol solution (1% (v/v) HCl in 70% ethanol) was added to differentiate the slices for 3–5 s. Slices were then immersed with eosin solution for 1 min. After washing, the slices were observed under an optical microscope.

After washing with PBS, the slices were incubated with 5% BSA for 2 h to block non‐specific sites for immunohistological analysis. Skin sections were incubated with Anti‐ Cytokeratin 10 (1:500 in PBS, Abcam, the USA), Anti‐Collagen IV antibody (1:200 in PBS, Abcam), and Anti‐Vimentin antibody (1:200, Sigma) respectively at 4 °C overnight. After washing three times using PBS (5 min each time), secondary antibodies (Alexa Fluor 561, 647, 488, Abcam) were added for 1 h at room temperature. Nuclei were stained with 2‐(4‐amidinophenyl)−6‐indolecarbamidine dihydrochloride (DAPI, Sigma) for 1 h at 6 µg mL^−1^, followed by washing three times with PBS. The stained skin sections were imaged using a Zeiss confocal microscope (Zeiss 710, Germany), and images were analyzed using ImageJ (the USA).

### UV irradiation on SEs

To closely mimic the conditions of native skin exposed to sunlight, the UVA irradiation system of the SE was established following a previous report.^[^ ^9^ ^]^ The UV lamp (Thorlabs, the USA) with the peak at 365 nm was fixed above a temperature‐controlled chamber, at a distance of 16 cm from the skin model. The temperature was maintained at 37 °C and the irradiation duration was 2.5 h. The total dose was 30 J cm^−2^, as this dose induces measurable deleterious effects.^[^ ^9^ ^]^

### ROS detection by fluorescence kit

To determine the effect of antioxidants, the production of ROS was used in cells as an indicator. The skin replicates (C/PCL/M 3) were first treated with antioxidants, namely, 1 mL 100 µg mL^−1^ Laa (Sigma, the USA), TPs (Medchemexpress, the USA), or both of them (Laa & TPs) in cell medium for 12 h. The samples in cell medium with and without UVA treatments were taken as the positive and negative control groups (abbreviated as PC and NC).

After 2.5 h of UV exposure, the artificial skin samples treated with antioxidants were washed with phosphate‐buffered saline (PBS, pH 7.4, Sigma). 1 mL 5 µm ROS detection kit (Thermo Fisher Scientific, the USA) prepared in the cell culture medium was added to each sample. The medium containing the ROS kit was removed after 30‐min incubation. Then the samples were fixed with 4% (PFA) for 15 min followed by washing three times with PBS. DAPI was applied to stain nuclei. The stained cells were sent for imaging with a confocal microscope mentioned before.

### Free radical detection by FNDs: Characterization of FNDs

Carboxylated 70 nm FNDs were obtained from Adámas Nano (Raleigh, NC, USA). The particles were produced by high‐pressure and high‐temperature synthesis followed by irradiation (with 3 MeV electrons and a fluence of 5 × 10^19^ e cm^−2^). After the annealing at the temperature above 600 °C, each particle contained ≈500 nitrogen vacancy centers (measured by the supplier) at average with a broad‐peak fluorescence over 600 nm.^[^ ^96^ ^]^ As a last step of the synthesis, the material was cleaned with oxidizing acids resulting in an oxygen‐terminated surface. These particles were extensively used and characterized in earlier literature already.^[^ ^97^ ^]^ The uptake of bare FNDs was measured in NHDF and HaCaT cells at 10, 30, and 50 µg mL^−1^. HaCaT and NHDF cells showed relatively low uptake of FNDs. In order to improve the uptake rate, the pure FNDs were coated with positively charged quaternized β‐chitin (QβC) that was synthesized following the previous study.^[^ ^98^ ^]^ The synthesis process and characterization are described in supporting information (Figure S1, Supporting Information).

To obtain the optimal uptake of FNDs, and maintain the cell metabolic activity, the concentrations of FNDs and QβC were optimized. In detail, 1 mg mL^−1^ FNDs were mixed with 5 mg mL^−1^ QβC in a cell medium, by adjusting the volume ratios, the final concentrations of FNDs were kept at 50 µg mL^−1^. QβC concentrations of 250 and 500 µg mL^−1^ were selected following the previous research.^[^ ^98^ ^]^ The particle size of the C/FNDs and the surface charge were determined via a zeta sizer (Malvern Panalytical Ltd, UK). The unmodified FNDs were measured as the control group.

To determine the morphology of coated and unmodified FNDs, TEM was used. TEM samples were deposited on a holey carbon film on 400 mesh copper TEM grids (Plano, Wetzlar, Germany, type, S147‐4) and dried under ambient conditions. To determine the agglomeration state of coated and unmodified FNDs, cryo‐TEM was used to avoid any drying‐related artifacts. Therefore, 2 µl of the sample solution was placed on a holey carbon grid, vitrified using a Gatan (Pleasanton, OR, USA) CP3 plunge freezer at −165 °C and transferred under liquid nitrogen to a Gatan model 914 cryo‐TEM sample holder operating at −170 °C. Bright‐field TEM images were acquired by a JEOL (Tokyo, Japan) JEM‐2100 LaB_6_ transmission electron microscope at 200 kV accelerating voltage and a Gatan Orius SC1000 CCD camera.

### Free radical detection by FNDs: Uptake and Biocompatibility of FNDs

After detachment using 0.5% w/v trypsin, HaCaT cells were seeded on 6‐well plates with 1 mL cell medium. For the different groups, this medium contained 50 µg mL^−1^ FNDs, 50 µg mL^−1^ FNDs, and 250 µg mL^−1^ QβC or 50 µg mL^−1^ FNDs and 500 µg mL^−1^ QβC, respectively. The same procedure was repeated for NHDF cells. To determine the biocompatibility of the diamonds, the metabolic activity of the cells was measured as follows. After 1‐day co‐incubation of FNDs and QβC, the cell metabolism was tested using an AlamarBlue kit following the protocol that was recommended by the manufacturer, and AlamarBlue reduction was calculated according to a previous study.^[^ ^99^ ^]^ Additionally, immunostaining was performed to show the FND uptake in NHDF and HaCaT cells. After incubation with diamonds for 1 day, cells were washed with PBS and fixed with 4% PFA for 10 min. After washing, the cells were treated with phalloidin‐FITC (Sigma, the Netherlands) for 45 min to label the f‐actin of the cytoskeleton. DAPI was used to stain cell nuclei. The samples were imaged by laser confocal microscopy (Leica Stellaris 5, Germany), and images were analyzed using ImageJ.

### Free radical detection by FNDs: Fabrication of Skin Models with C/FNDs and ROS Detection Using T1 Relaxometry

To quantify the ROS production in the dermal layer, 50 µg mL^−1^ FNDs coated with 250 µg mL^−1^ QβC were chosen to do diamond uptake in NHDF cells. The cells at the bottom of the flasks were washed with PBS and detached using trypsin. The skin models with FNDs were fabricated using the protocol described in 2.2.1. These skin models (M/C/PCL 3) were treated with antioxidants and irradiated with a UV lamp as depicted in 2.3 after 3‐day co‐culture. T1 measurements were performed with a home‐built relaxometer which was described earlier.^[^ ^100^, ^101^ ^]^ In short, the equipment is a home‐built confocal microscope with the following specifics. A 532 nm laser (50 µW laser power in the place of the sample, measured under continuous illumination) was used. Using this laser and an acousto‐optical modulator (Gooch & Housego, model 3350‐199) the spin states of NV centers were able to manipulate and measure in diamond were manipulated and measured. To this end, the NV centers were irradiated for 5 µs which pumped into the bright ms = 0 state of the ground state. Then they were left in the dark to relax back to the equilibrium between ms = 0 and ms = + −1 at different times (0.2 µs–10 ms). For a satisfactory signal‐to‐noise ratio, the pulsing sequence was repeated 10 000 times for each measurement. In the presence of paramagnetic species (in this case free radicals), this process occurs faster.^[^ ^102^ ^]^ To assess the impact of the FND modification on T1 signals, 10 µm gadolinium in aqueous solution was used. Then the T1 experiments were performed on the FNDs in skin samples before and after UV exposure. The relaxation time was fitted by the biexponential function below (Equation (1)), and the longer‐time values were summarized as the T1 constants.^[^ ^93^ ^]^

(1) $$PL\left(\tau \right)\;=\;I_{\inf}\;\left(1\;+\;C_{a}\;e^{-\tau /Ta}+C_{b}\;e^{-\tau /Tb}\right)$$

### Free radical detection by FNDs: Calculation of ROS Concentrations from T1

To estimate the free radical concentrations from T1 values, the measurements were calibrated with known concentrations of radicals as shown in.^[^ ^93^ ^]^ More specifically, the study quantified *OH which were generated by UV irradiation of H_2_O_2_. For the relationship between *T1* and C*_OH_ (concentration of *OH), it is found that the following exponential equation^[^ ^37^, ^93^ ^]^

(2) $$T1\left(C_{*\mathrm{OH}}\right)\;=\;T1_{\infty }\;\left(1\;+\;A\;e^{-C_{*\mathrm{OH}}/C_{0}\;}\right)$$

with *T*1_∞_ in µs, *C*_0_ in µm, and *A* is dimensionless.

The parameters of the model in Equation (1) was calculated by a nonlinear fitting procedure.

(3) $$T1_{\infty }=116.6\pm 1.05\;\mu s$$

(4) A=1.5±0.03

(5) $$C_{0}\;=\;0.12\pm 0.009\;\mu M$$

### Statistical Analysis

*T1 data analysis*: The T1 raw data were processed by MATLAB (the USA), and the outliers were removed by calculating with the Outlier Calculator (MiniWebtool). The statistical analysis was performed using the Unpaired t‐test with Welch's correction in Graphpad prism, to determine the presence of significant differences among the untreated and antioxidant‐treated groups.

*Other data analysis*: The statistical analysis of other data presented in this manuscript was performed using a one‐way Analysis of Variance (ANOVA), and multiple comparisons. This analysis was conducted utilizing GraphPad Prism.

The results in the manuscript were presented as Mean ± standard deviation (SD), calculated from numerous data points across multiple experimental conditions. Sample sizes were provided in the captions of the corresponding figures.

## Conflict of Interest

The authors declare no conflict of interest.

## Supporting information

Supporting Information

## Data Availability

The data that support the findings of this study are available from the corresponding author upon reasonable request.
