# Supplementary material for: Quantum Sensing Unravels Antioxidant Efficacy Within PCL/Matrigel Skin Equivalents
Source: Small. 2024 Sep 9;20(49):2403729. doi: 10.1002/smll.202403729 (PMC11618742; doi:10.1002/smll.202403729)
Supplement: Supplementary file 1 — Supporting Information [file SMLL-20-2403729-s001.docx]

Quantum Sensing Unravels Antioxidant Efficacy within PCL/Matrigel Skin Equivalents

Xixi Wu^a^, Marcus Koch^b^, Felipe P. Perona Martínez^a^, Romana Schirhagl^a,*^, Małgorzata K. Włodarczyk-Biegun^c, d, *^

^a^ Department of Biomedical Engineering, University Medical Centre Groningen and University of Groningen, Ant. Deusinglaan 1, 9713 AV Groningen, The Netherlands

^b^ INM - Leibniz Institute for New Materials, Campus D2 2, 66123 Saarbruecken, Germany

^c^ Polymer Science, Zernike Institute for Advanced Materials, University of Groningen, Nijenborgh 4, 9747 AG, The Netherlands

^d^ Biotechnology Centre, The Silesian University of Technology, Krzywoustego 8, 44-100 Gliwice, Poland

*Corresponding author: Romana Schirhagl, Małgorzata K. Włodarczyk-Biegun

E-mail: [r.schirhagl@umcg.nl](mailto:r.schirhagl@umcg.nl), m.k.wlodarczyk@rug.nl

**Materials and methods**

**Synthesis and** **characterization of quaternized β-chitin (Q****βC)**

The QβC was synthesized in our previous study according to Xu *et al.* research. ^[1]^ β-Chitin was extracted from squid pens. The initial β-chitin was purified with 1 M aqueous NaOH overnight for deproteination and then with 1 M aqueous HCl overnight for demineralization at room temperature. The obtained β-chitin was washed with deionized water between each step and the purification procedures were repeated twice. Then the purified β-chitin was lyophilized and ground into powder. To make QβC, 20 g KOH and 4 g urea were dissolved in 75 mL distilled water at -20°C. Then, 1 g β-chitin powder was added immediately to the solution, followed by vigorous stirring for 30 min to obtain a transparent solution. 16 g of 2, 3-epoxypropyl trimethylammonium chloride (EPTMAC) (Sigma, the USA) were added dropwise into 100 g of β-chitin solution, the mixture was stirred vigorously at 0°C for 24 hours. After that, the mixture was neutralized with aqueous 5 M HCl, dialyzed (MWCO≈3500 Da) against distilled water for 7 days and then lyophilized. ^1^H-NMR (Bruker Ascend 600 FT-NMR, USA) and FTIR (Bruker IFS88, USA) were applied to determine the structure of QβC, and the surface charge was determined via zeta potential measurements (Malvern Panalytical Ltd, UK).


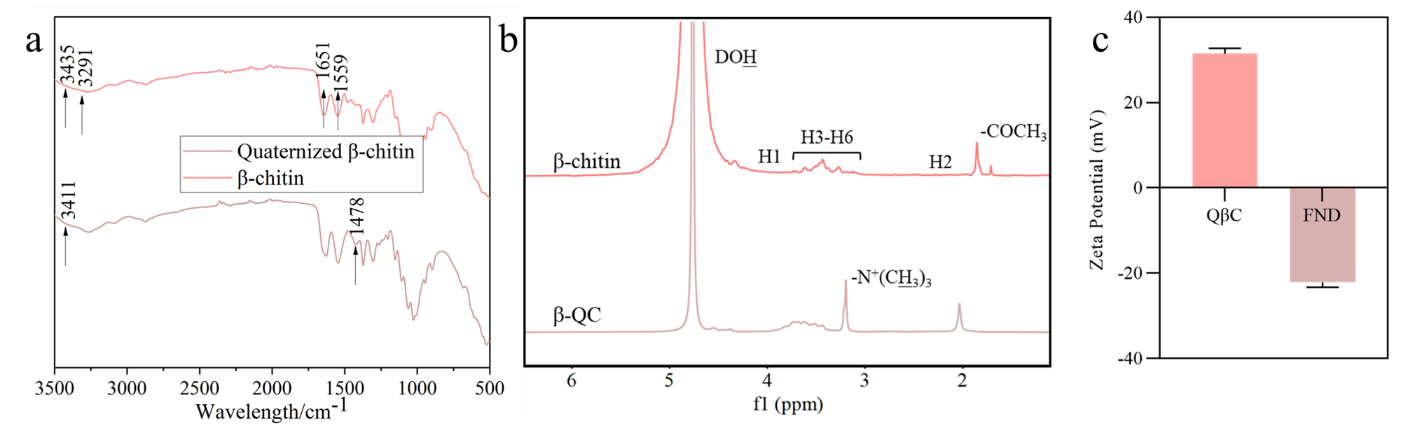
Figure S1. Physicochemical characterization of materials. a) FTIR spectrum and b) ^1^H-NMR spectrum of QβC and untreated β-chitin. c) Zeta potential of QβC and FNDs.

To confirm the chemical structure of QβC, FTIR and ^1^H-NMR characterizations were performed (see Fig. S1a and b). The FTIR spectrum of the purified β-chitin contained the absorption peaks at 3435 cm^−1^ for OH stretching, 3291 cm^−1^ for NH stretching, 1651 cm^−1^ for the amide I band (υ(C=O)) and 1559 cm^−1^ for the amide II band (δ(N–H)). After quaternization, the OH stretching absorption peak shifted from 3435 to 3411 cm^−1^, and the new characteristic absorption peak of the C–H bending of –N^+^(CH_3_)_3_ showed at 1478 cm^−1^, indicating the existence of quaternary ammonium groups in QβC. This grafting result was also consistent with the ^1^H spectra of QβC using ^1^H-NMR analysis. QβC and β-chitin were dissolved in NaOD (Sigma) solution. The hydroxyl protons of the N-acetyl-glucosamine residue contributed to the DOH signal produced by deuteration with NaOH/D_2_O. The signals of protons in the glucopyranose ring ranged from 2.23 to 3.80 ppm, and the protons of the –N^+^(CH_3_)_3_ group appeared in the new peak at 3.18 ppm, confirming the formation of quaternized β-chitin. The methyl protons of the acetamido groups appeared at 1.85 and 1.72 ppm, while the protons from QβC located at 2.02 ppm. That might be caused by esterification between chitin.

The analysis of FTIR and NMR spectra proved that the β-chitin was successfully modified by the EPTMAC, which was validated previously. ^[2–4]^ The zeta potential of QβC was 36.5 ± 0.5 mV, and zeta potential of the FND was -22.5 ± 1.3 mV (Fig. S1c). Thus, we envisioned that the coating of QβC on FNDs can be stabilized by electrostatic attraction between QβC and NDs.

**The density and porosity measurement on PCL scaffolds**

The liquid displacement technique was used to measure the density (g/cm^3^) of the scaffolds according to the methods described in a previous study. ^[5]^ PBS was used as displacement liquid. The weight of scaffolds (W) was measured and the material was dipped into a known volume (V1) of PBS in a measuring cylinder for 5 min. The total volume of PBS and PBS-impregnated scaffold was recorded as V2. Subsequently, the scaffold was removed from the measuring cylinder, and the remaining volume of PBS in the measuring cylinder was recorded as V3. By using the equations below, the density and porosity of the scaffolds were estimated.

Total volume of the scaffold: V2 (1)

Density of the scaffold: D = W/(V2 – V3) (2)

Porosity of the scaffold was: P = (V1 – V3)/(V2 – V3) (3)

**Weight loss and swelling ratio study on PCL/Matrigel composites**

For the degradation study, the PCL/Matrigel composites were prepared, dried, and weighed, and the initial weight of dry samples was noted as W0. After keeping the samples in PBS at 37°C for 1, 3, and 7 days, the PBS solution was removed, the composites were dried, then weighed, and the final weights were noted as W1. The ratio of weight loss was calculated using the following equation:

Weight loss ratio = (W0-W1)/W0 *100%

A swelling study was also conducted following previous research. ^[6]^ The PCL/Matrigel composites were prepared as described previously, then the samples were lyophilized overnight. The dry composites were weighed, and the initial weight of each composite was noted as W0. The composites were then transferred into one-chamber Petri dishes, immersed in 1 mL PBS, and kept at 37°C. After 1 hour, 1 day, 3 days, and 7 days, the PBS was removed carefully, and the samples were weighed again, the weights of them were noted as W1. The swelling ratios of the samples were calculated by the following equation:

Swelling ratio = (W1 – W0)/W0 *100%

The results of weight loss and swelling ratio were shown in Fig. SI 6.

The liquid displacement technique was used to measure the density (g/cm^3^) of the scaffolds according to the methods described in a previous study. ^[5]^ PBS was used as displacement liquid. The weight of scaffolds (W) was measured and the material was dipped into a known volume (V1) of PBS in a measuring cylinder for 5 min. The total volume of PBS and PBS-impregnated scaffold was recorded as V2. Subsequently, the scaffold was removed from the measuring cylinder, and the remaining volume of PBS in the measuring cylinder was recorded as V3. By using the equations below, the density and porosity of the scaffolds were estimated.

Total volume of the scaffold: V2 (1)

Density of the scaffold: D = W/(V2 – V3) (2)

Porosity of the scaffold was: P = (V1 – V3)/(V2 – V3) (3)


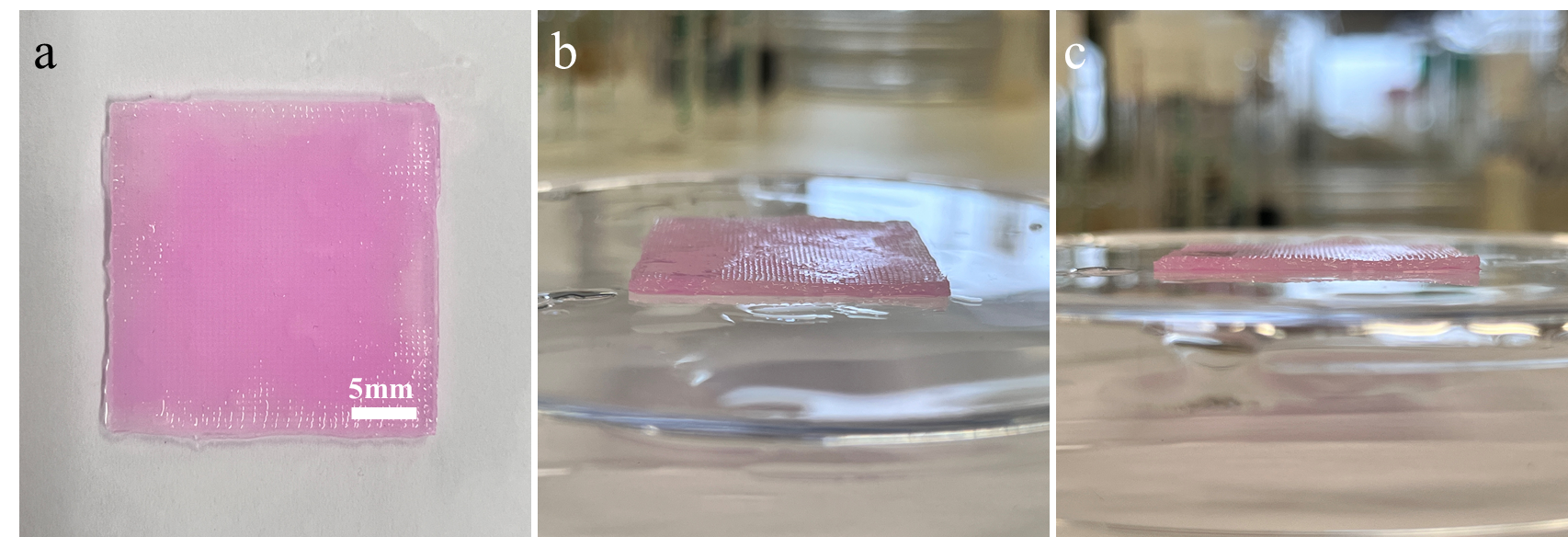


Figure S2.a-c) Optical images of PCL/Matrigel composites. The red color is originated from the phenol red in Matrigel. Scale bar is shown in the figure.


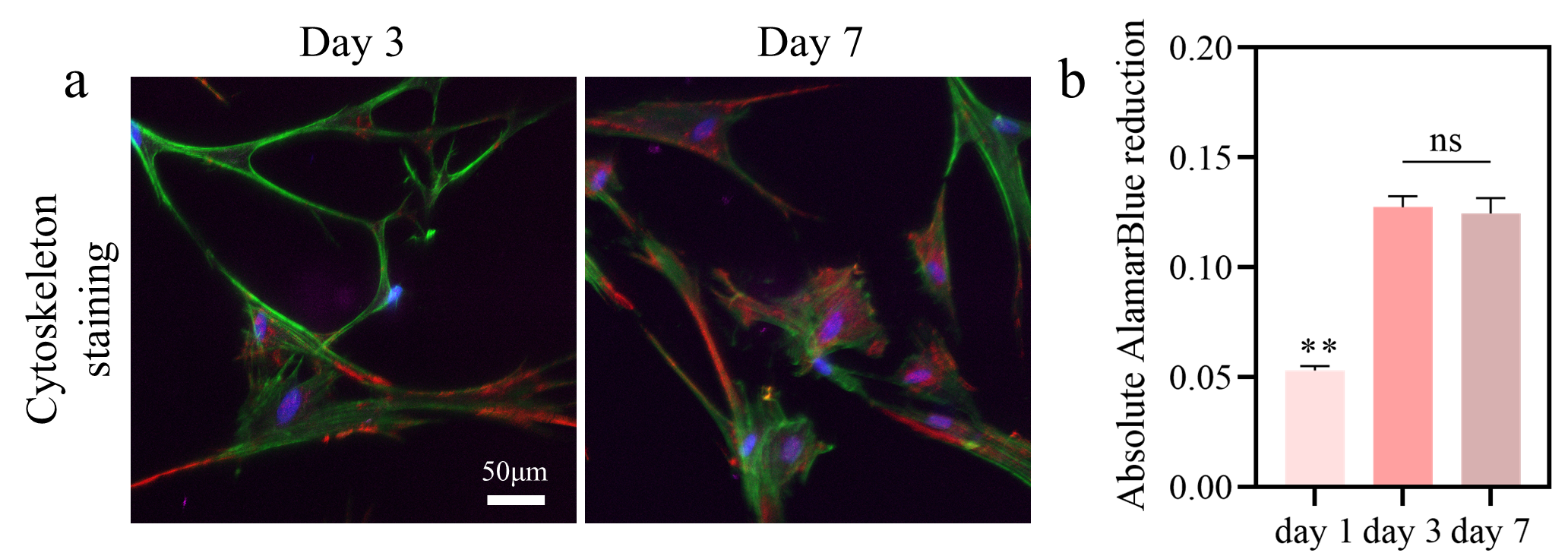


Figure S3. a) NHDF cell growth in Matrigel. Spindle-shaped cells were observed clearly in Matrigel after 3-day and 7-day cultures. The f-actin was in green, vimentin was labeled in red, Collagen I was marked in magenta, the cell nuclei were in blue. 3 samples of each experimental group were assessed. 5-6 views of each sample were observed under the confocal microscope. b) The cell metabolism of the 3D-cultured cells in Matrigel at days 1, 3, and 7 as indicated by the absolute AlamarBlue reduction. The statistical significance was done using the ANOVA test, ** indicates p < 0.01. Scale bar: 50 μm. 20 images of cell nuclei were used to evaluate the mentioned cell behavior.


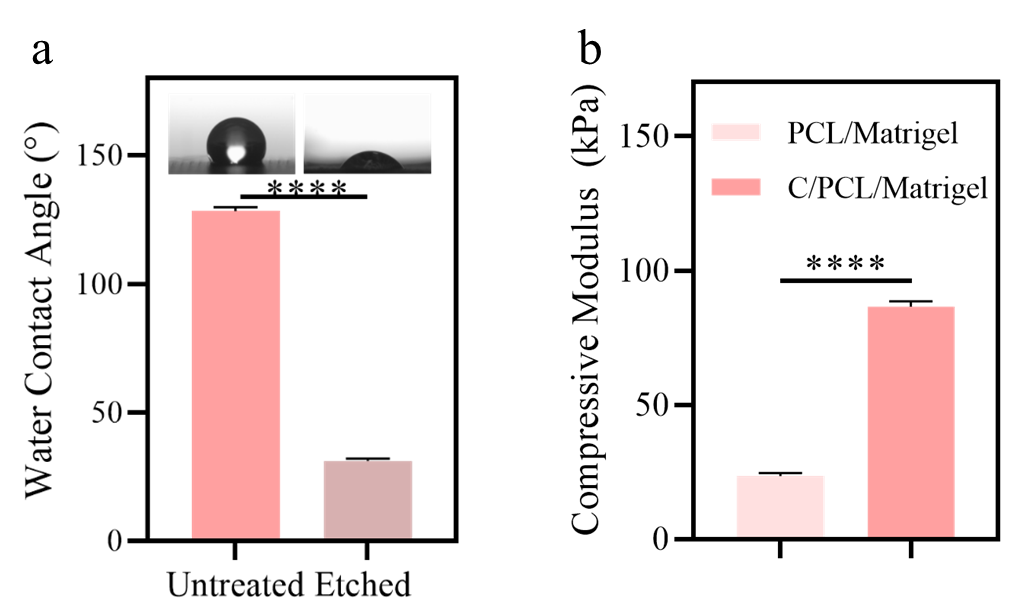


Figure S4. The water contact angle of the untreated PCL scaffolds and alkali-treated (etched) scaffolds. The statistical significance was analyzed using the T-test, with **** indicating p < 0.0001.


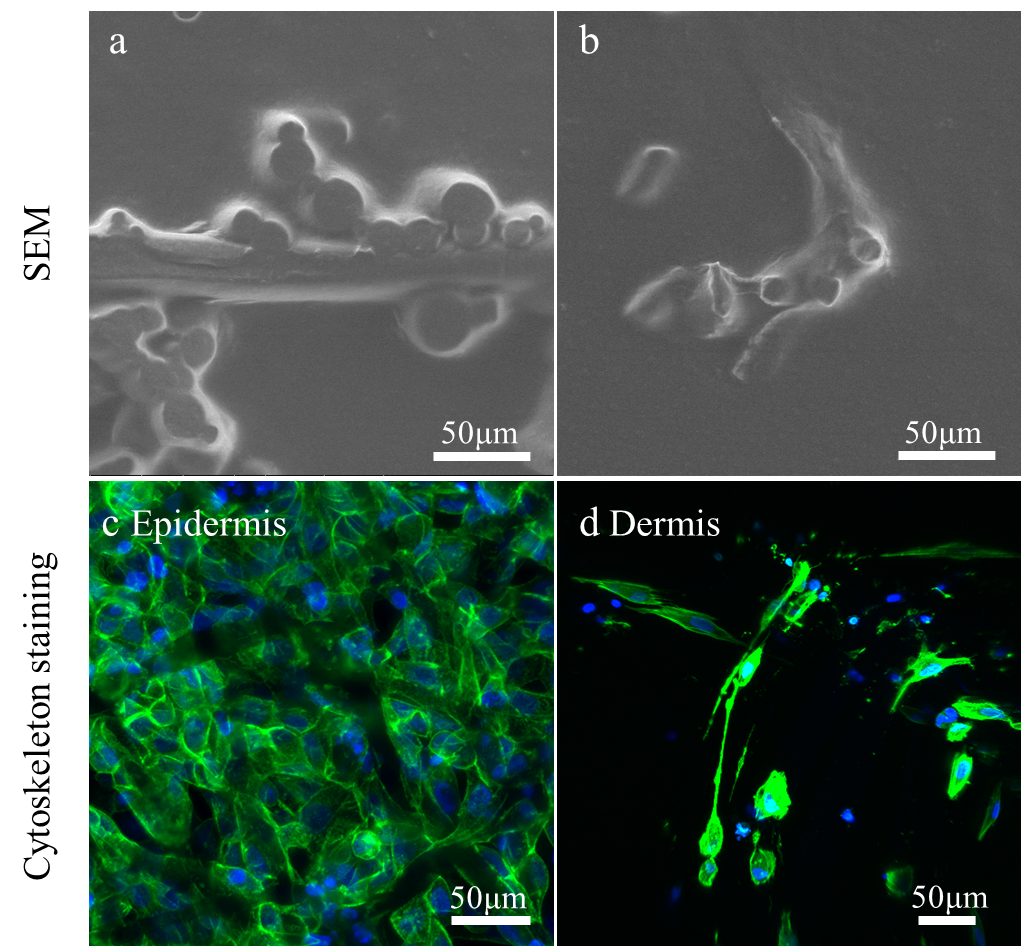


Figure S5. SEM images of cell-laden PCL/Matrigel slices: a) epidermis; b) dermis. c) Fluorescent images of cells in PCL/Matrigel composites. F-actin was labeled in green, cell nuclei were stained in blue. 3-4 samples were observed, 3 views were captured and analyzed. Scale bar: 50 μm.


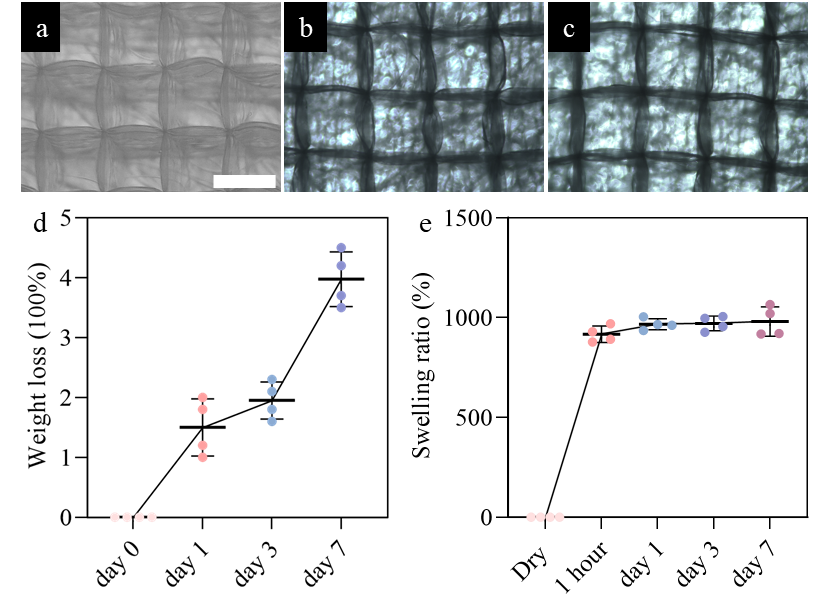


Figure S6. a-c) Optical images of PCL/Matrigel composites on days 1, 3, and 7 after incubation at 37°C in PBS. No obvious scaffold deformation or morphology change of the composites was observed. Scale bar: 500 μm. d) Weight loss shows little degradation of the composites (less than 5 wt.%) within one week, and e) swelling ratios after 1 hour, 1 day, 3 days, and 7 days of incubation at PBS. 4 samples of each experimental group were evaluated, and mean values with SD were presented.


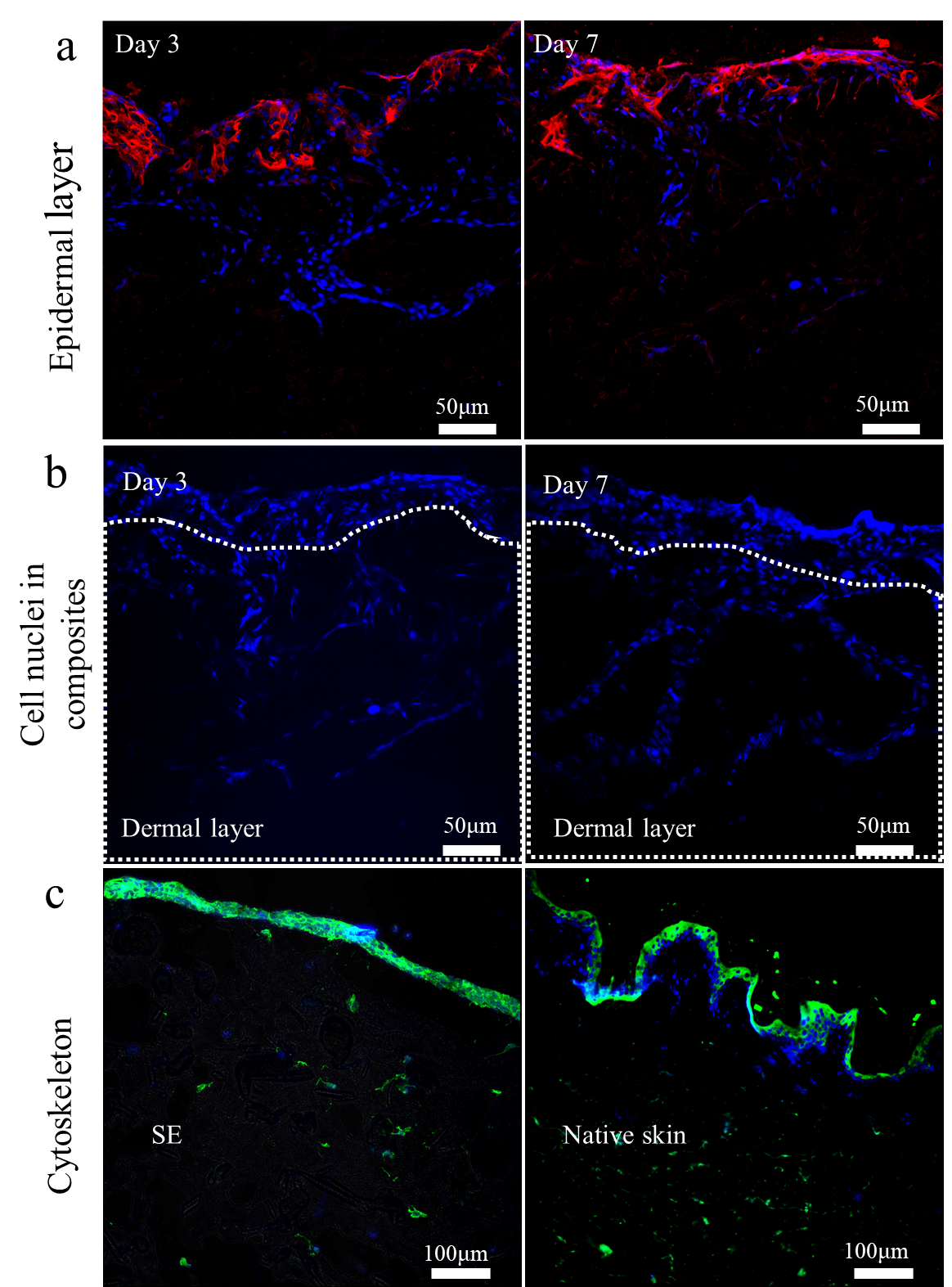


Figure S7. a) SEs after 3 and 7 days’ culture, the epidermal layer is labelled with keratin biomarker in red and cell nuclei in blue. b) The homogeneous cell distribution and penetration in SEs are indicated by the cell nuclei in blue. c) The cell nuclei staining shows the cell distribution, penetration depth, cell migration, and proliferation at 3-day coculture. Cells are homogeneously dispersed in the dermal layer. The cell penetration depth (ca. 700 μm) and the numbers of cell nuclei were 37 ± 10 (day 3), and 42 ± 8 (day 7) respectively. No obvious cell migration was observed between the epidermal and dermal layers. Scale bars are shown in the images.


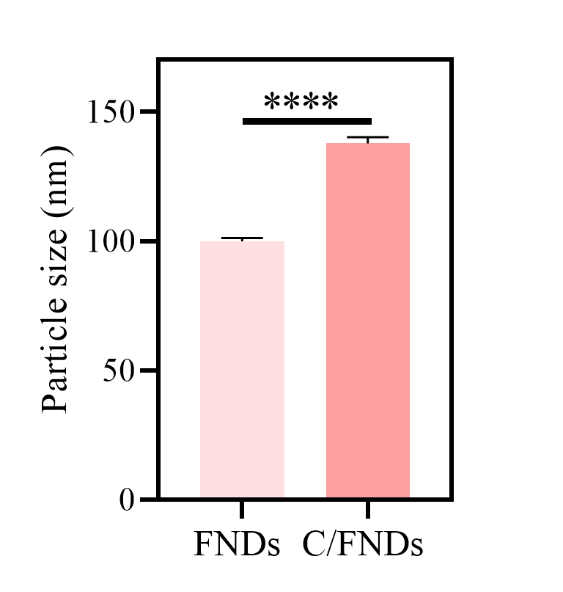


Figure S8. Particle sizes of bare FNDs and C/FNDs defined by zetasizer instrument. The statistical significance was analyzed using the ANOVA test, with **** indicating p < 0.0001.


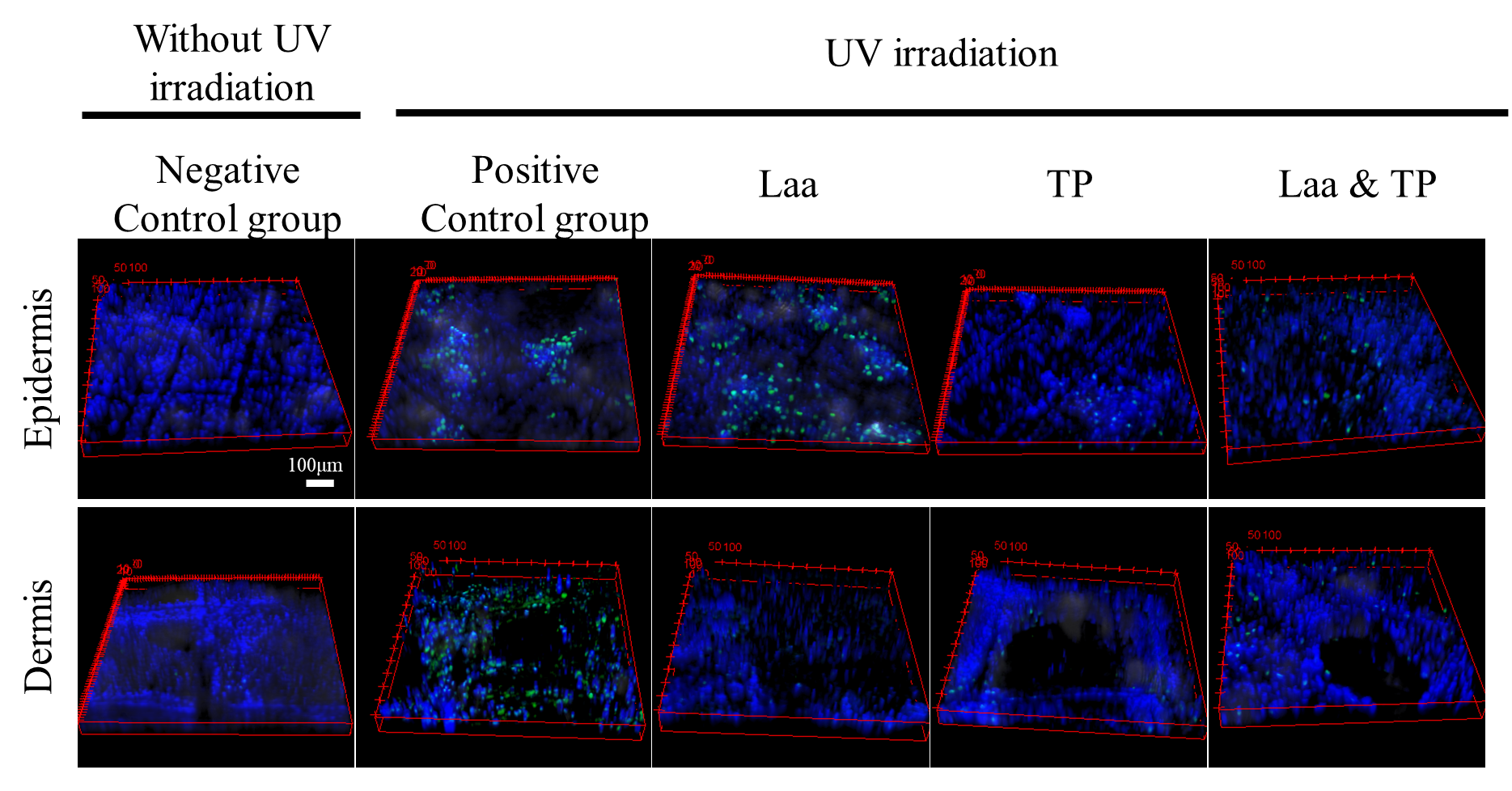


Figure S9. 3D fluorescent staining of SE showing the production of ROS in epidermal and dermal cells before and after UVA radiation. Negative control for untreated SEs, the UVA-irradiated SEs in normal cell medium were used as a positive control. The experimental SEs were treated with Laa, TP, Laa&TP respectively. ROS were stained with green fluorescence, and cell nuclei were labeled in blue. 3 samples of each experimental group were analyzed. Scale bar: 100 μm.


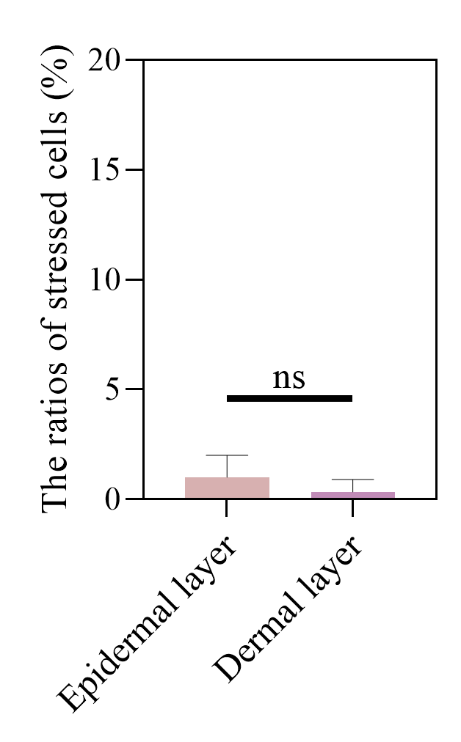


Figure S10. The ratios of stressed cells in negative control groups. The low ratios (1.0%, 0.67% individually) show that the impact of factors other than UVA irradiation can be neglected. The experiment was repeated 3 times, and error bars represent the standard deviations. Statistical comparisons were made using the ANOVA test, with ns indicating no signiﬁcant diﬀerence.


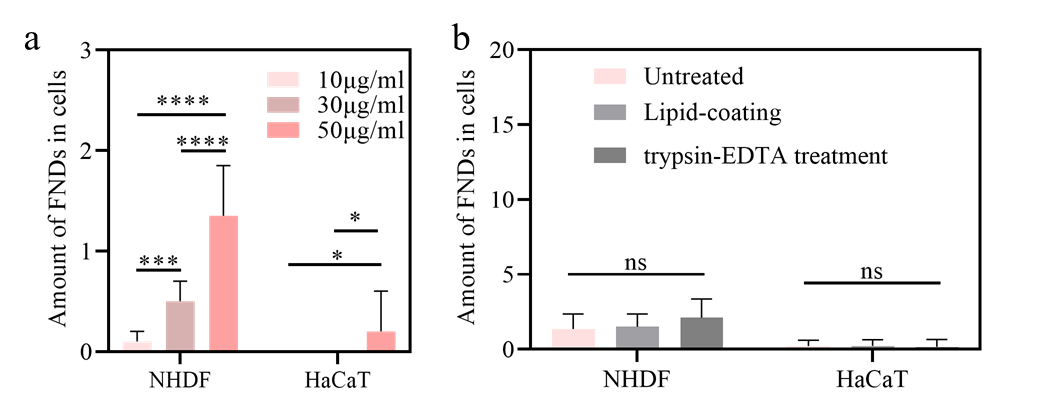


Figure S11. a) Bare FND uptake in untreated NHDF and HaCaTs at different diamond concentrations. b) Diamond uptake in NHDF and HaCaTs after lipid coating and treatment with trypsin-EDTA to cells at the same FND concentration (50 μg/mL). The untreated group means no treatment to cells and no coating of FNDs. The group is used as a control. The statistical significance was analyzed using the ANOVA test, ns means no significant difference, * means p<0.05, *** means p<0.001, and **** means p<0.0001.


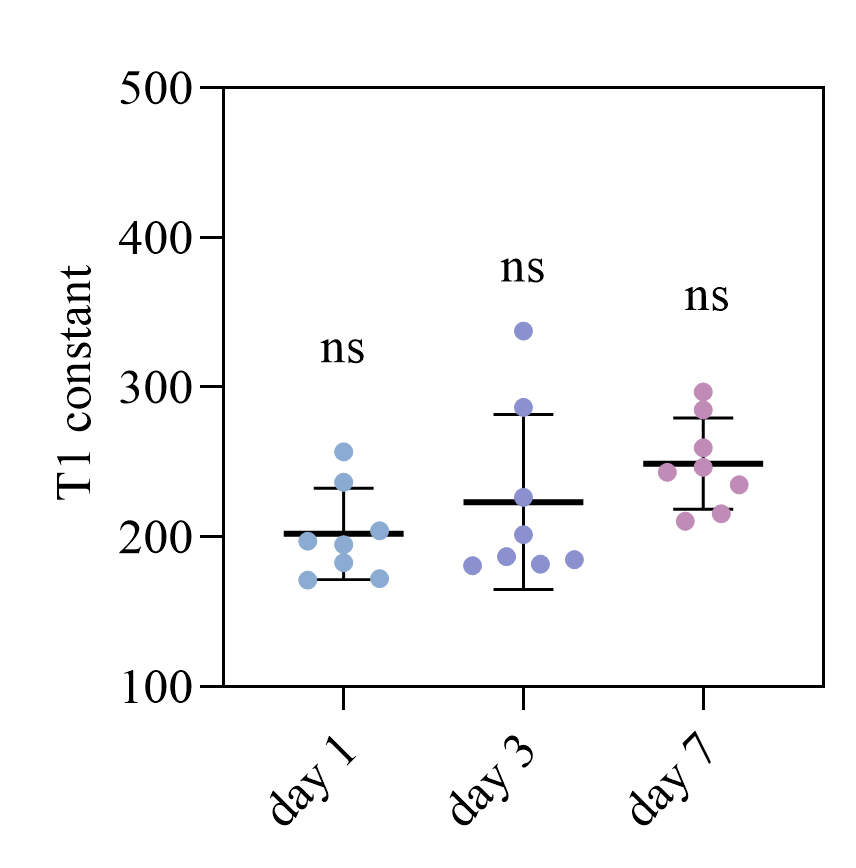


Figure S12. T1 measurement of PCL/Matrigel during one-week incubation. 7 FNDs were tested in each experimental group, no significant difference was found among days 1, 3, and 7. The statistical significance was analyzed using a One-way ANOVA test, ns means no significant difference.


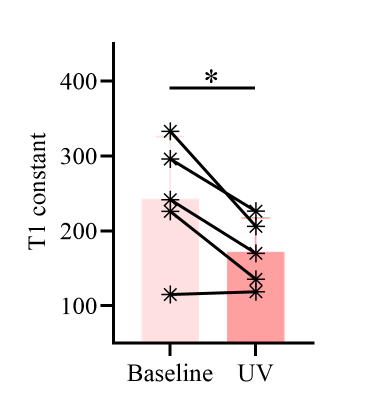


Figure S13. T1 measurements in SEs cultured after 7 days. A decrease in T1 values was observed after treatment with UVA irradiation. Significant difference was analyzed by a paired T-test, here * indicates p<0.05.

Reference

[1] H. Xu, Z. Fang, W. Tian, Y. Wang, Q. Ye, L. Zhang, J. Cai, *Advanced Materials* **2018**, *30*, DOI 10.1002/adma.201801100.

[2] A. G. B. Pereira, E. C. Muniz, Y. Lo Hsieh, *Carbohydr Polym* **2015**, *123*, DOI 10.1016/j.carbpol.2015.01.017.

[3] H. Xu, L. Zhang, J. Cai, *ACS Appl Bio Mater* **2019**, *2*, DOI 10.1021/acsabm.8b00548.

[4] W. Hu, Z. Wang, Y. Zha, X. Gu, W. You, Y. Xiao, X. Wang, S. Zhang, J. Wang, *Adv Healthc Mater* **2020**, *9*, DOI 10.1002/adhm.202000035.

[5] S. K. J. Ufere, N. Sultana, *J Teknol* **2016**, *78*, DOI 10.11113/jt.v78.10072.

[6] N. T. Lam, H. Lam, N. M. Sturdivant, K. Balachandran, *Biomedical Materials (Bristol)* **2017**, *12*, DOI 10.1088/1748-605X/aa71be.
